# High correlation of hepatic shear wave velocity with esophageal varices complication rate in patients with chronic liver diseases

**DOI:** 10.1186/s12876-023-02821-4

**Published:** 2023-05-22

**Authors:** Shouichi Namikawa, Takuto Nosaka, Hidetaka Matsuda, Yu Akazawa, Kazuto Takahashi, Tatsushi Naito, Masahiro Ohtani, Yasunari Nakamoto

**Affiliations:** Second Department of Internal Medicine, Faculty of Medical Sciences, University of Fukui, 23-3 Matsuoka Shimoaizuki, Eiheiji-Cho, Yoshida-Gun, Fukui, 910-1193 Japan

**Keywords:** Esophageal varices, Shear wave velocity, Chronic liver disease

## Abstract

**Background:**

Histological evaluation by liver biopsy is considered the gold standard for assessing liver disease; however, it is highly invasive. Non-invasive liver stiffness measurement by shear wave elastography (SWE) is effective for evaluating the hepatic fibrosis stage and related diseases. In this study, we investigated the correlations of liver stiffness with hepatic inflammation/fibrosis, functional hepatic reserve, and related diseases in patients with chronic liver disease (CLD).

**Methods:**

Shear wave velocity (Vs) values were measured using point SWE in 71 patients with liver disease from 2017 to 2019. Liver biopsy specimens and serum biomarkers were collected at the same time, and splenic volume was measured using computed tomography images with the software Ziostation2. Esophageal varices (EV) were evaluated by upper gastrointestinal endoscopy.

**Results:**

Among CLD-related function and complications, Vs values were highly correlated with liver fibrosis and EV complication rates. The median Vs values for liver fibrosis grades F0, F1, F2, F3, and F4 were 1.18, 1.34, 1.39, 1.80, and 2.12 m/s, respectively. Comparison of receiver operating characteristic (ROC) curves to predict cirrhosis showed that area under the ROC (AUROC) curve for Vs values was 0.902, which was not significantly different from the AUROCs for the FIB-4 index, platelet count, hyaluronic acid, or type IV collagen 7S, while it was significantly different from the AUROC for mac-2 binding protein glycosylation isomer (M2BPGi) (*P* < 0.01). Comparison of ROC curves to predict EV showed that the AUROC for Vs values was 0.901, which was significantly higher than the AUROCs for FIB-4 index (*P* < 0.05), platelet count (*P* < 0.05), M2BPGi (*P* < 0.01), hyaluronic acid (*P* < 0.05), and splenic volume (*P* < 0.05). In patients with advanced liver fibrosis (F3 + F4), there was no difference in blood markers and splenic volume, while Vs value was significantly higher in patients with EV (*P* < 0.01).

**Conclusions:**

Hepatic shear wave velocity was highly correlated with EV complication rates in chronic liver diseases as compared to blood markers and splenic volume. In advanced CLD patients, Vs values of SWE are suggested to be effective in predicting the appearance of EV noninvasively.

## Background

Chronic liver disease (CLD) is a major public health problem, accounting for significant morbidity and mortality worldwide [1, 2]. Cirrhosis develops after a long period of chronic inflammation such as hepatitis B or C virus infection, alcoholism, and nonalcoholic steatohepatitis that results in replacement with fibrotic tissue, leading to portal hypertension [3, 4]. Histological evaluation by liver biopsy is considered the gold standard for assessment of the nature and severity of liver diseases, including liver fibrosis and vascular structures [2, 5, 6]. However, liver biopsy is a highly invasive and inappropriate test for monitoring the progression of liver disease [2].

In recent years, non-invasive tests have become an area of research in liver disease [2, 7]. Shear wave elastography (SWE), a non-invasive test, is a technique that measures liver stiffness by quantifying the velocity of induced shear waves [8]. The liver stiffness values have been reported to reflect the hepatic fibrosis stage [9], functional hepatic reserve [10, 11], and complications related to portal hypertension [12]. However, which of these CLD-related diseases and functions are highly correlated with stiffness has not been fully assessed. In addition, the diagnostic accuracy of liver stiffness has not been sufficiently validated with histological evaluation by liver biopsy, serum biomarkers, or spleen volume.

In this study, we investigated the correlations of liver stiffness measured by point SWE (pSWE) with hepatic inflammation/fibrosis stage, functional hepatic reserve, and portal hypertension-related complications in patients with CLD. Not only did pSWE values correlate with the stages of fibrosis assessed by liver biopsy and non-invasive tests such as serum biomarkers and splenic volume, but interestingly, the values were highly correlated with the appearance of esophageal varices (EV) in patients with advanced fibrotic liver diseases.

## Methods

### Patients

We reviewed data of 71 patients with liver disease who underwent blood biochemical tests, assessment of shear wave velocity (Vs) using SWE, and liver biopsy at the Fukui University Hospital between April 2017 and March 2019. The background data of the patients are summarized in Table 1. Blood tests performed on the day of admission included platelet count and serum levels of aspartate aminotransferase (AST), alanine aminotransferase (ALT), alkaline phosphatase (ALP), bilirubin, albumin, international normalized ratio (INR), mac-2 binding protein glycosylation isomer (M2BPGi), hyaluronic acid, and type IV collagen 7S. Subsequently, a liver biopsy was performed. Of the 71 patients, 45 patients underwent upper gastrointestinal endoscopy within one year before or after the date of SWE. Endoscopic findings related to EVs are summarized in Table 1. This study was conducted in accordance with the Declaration of Helsinki. The study design was approved by the Research Ethics Committee of the University of Fukui (registration number 20170016).

**Table 1:** Patient characteristics

Age, years	65	(55–72)
Sex, female/male	43/28	
Etiology, HBV/HCV/NASH/Alcohol/AIH/PBC/Others	4/26/9/5/8/8/11	
Aspartate aminotransferase, IU/L	46	(31–90)
Alanine aminotransferase, IU/L	43	(26–97)
Gamma-glutamyl transpeptidase, IU/L	82	(33–153)
Alkaline phosphatase, IU/L	329	(236–452)
Albumin, g/dL	4	(3.6–4.3)
Total bilirubin, mg/dL	0.8	(0.6–1.2)
Total cholesterol, mg/dL	178	(146–212)
Fasting plasma glucose, mg/dL	94	(88–115)
Platelet count, × 10^3^ mm3	187	(129–242)
Prothrombin time, international normalized ratio	1.01	(0.97–1.09)
M2BPGi, C.O.I	2.07	(1.19–4.23)
Hyaluronic acid, ng/mL	93.6	(44.1–239)
Type IV collagen 7S, ng/mL	5.7	(4.3–8.1)
FIB-4 index	3.03	(1.70–4.32)
Fibrosis stage, F0/F1/F2/F3/F4	7/14/21/19/10	
Esophageal varices, -/ +	35/10	
Esophageal varices Form, 1/2/3	6/4/0	
Esophageal varices Color, White/Blue	1/9	
Esophageal varices Red color sign, -/ +	6/4	

Values are expressed as n or median (interquartile range)

*HBV* hepatitis B virus, *HCV* hepatitis C virus, *NASH* non-alcoholic steatohepatitis, *AIH* autoimmune hepatitis, *PBC* Primary Biliary Cholangitis, *M2BPGi* mac-2 binding protein glycosylation isomer

### Evaluation of liver stiffness

Abdominal ultrasonography was performed using Hitachi HI VISION Ascendus (Hitachi Medical Corporation, Tokyo, Japan) to estimate the shear wave measurement (SWM). Within a single SWM measurement, several push track sequences are delivered, and the SWM samples Vs in multiple positions at different depths within the region of interest (ROI). This is automatically repeated within a short time (< 1 s). Based on the acquired SWM, the system displays a histogram and overview. The distribution of Vs is displayed in the histogram, interquartile range (IQR), depth of the sample, median Vs (m/s), and transformed kPa. This method has a built-in feature, the VsN, which is the reliability index of the Vs acquired per measurement and functions as a quality indicator; it ranges from 0–100% [13, 14]. In this study, SWM was measured at least three times in the right lobe of the liver and the average of two Vs values was adopted from those with higher VsN values.

### Histological evaluation

Liver biopsy specimens were obtained percutaneously from the right lobe of the liver using a 16G or 18G biopsy needle under ultrasound guidance. Tissue samples were fixed in formalin, embedded in paraffin, and stained with hematoxylin–eosin, Masson’s trichrome, and Azan stains. A pathologist evaluated the specimens histologically. Liver fibrosis was staged according to the new Inuyama classification [15].

### Evaluation of EVs by upper gastrointestinal endoscopy

Endoscopy was performed within one year before and after the date of SWE. Upper gastrointestinal endoscopy was performed using GIF-H290Z (Olympus, Tokyo, Japan) endoscope. The endoscopic findings of EVs were evaluated according to the grading system outlined in the General Rules for Recording Endoscopic Findings of Esophagogastric Varices (2nd edition) prepared by the Japan Society for Portal Hypertension [16].

### Serum biochemical marker assays

Blood platelet count and serum levels of M2BPGi, hyaluronic acid, and type IV collagen 7S were measured on the day of liver biopsy as indicators of liver fibrosis. We also determined the parameters that allowed us to calculate the FIB-4 index using the following formula: (age [years] × AST [IU/L]) / (platelets [10^3^/mm^3^] × ALT [IU/L]^1/2^) [17].

### Measurement of splenic volume

Splenic volume was measured using the commercially available software Ziostation2 (ziosoft inc., Tokyo, Japan). The spleen identified on the computed tomography (CT) images was reconstructed in three dimensions with Ziostation2, and the spleen volume was calculated.

### Statistical analysis

One-way analysis of variance, receiver-operating characteristic (ROC) curve analysis, and Mann–Whitney’s U test were used to evaluate the results. Correlations were evaluated using Spearman's rank correlation coefficient. Statistical significance was defined as *P* < 0.05. Statistical analyses were performed using Prism software (version 9.2.0; GraphPad, GraphPad Software Inc., San Diego, CA, USA) and EZR software program (version 1.61; Saitama Medical Center, Jichi Medical University, Saitama, Japan). Youden's index (sensitivity + specificity—1) was used to identify the optimal cutoff point.

## Results

### Vs value highly correlated with liver fibrosis and esophageal varices

**Fig. 1:**
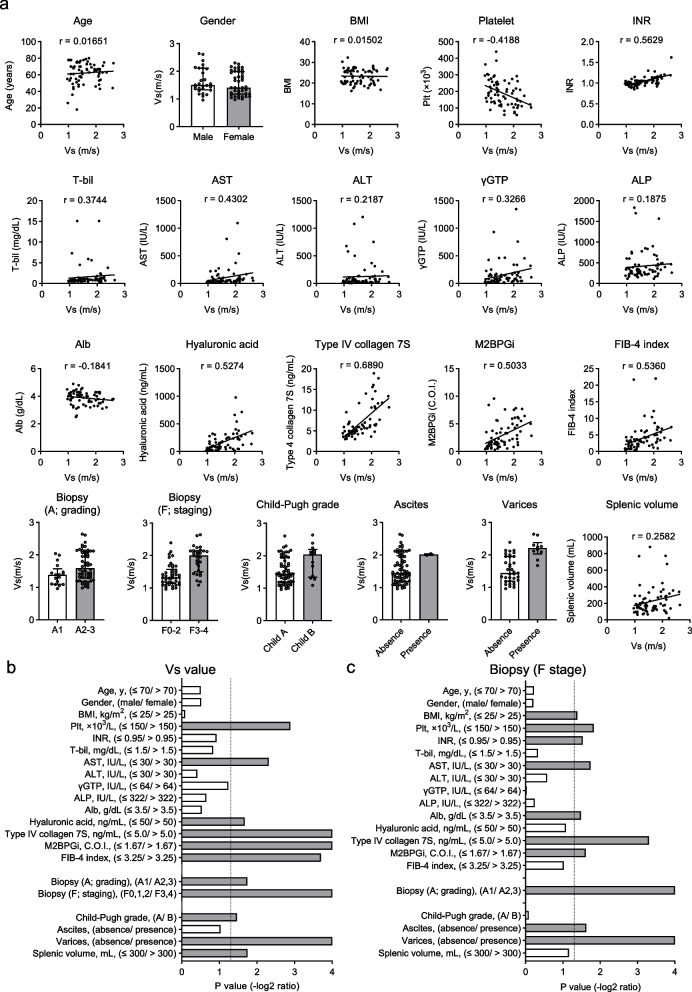
The correlation between Vs values measured by shear wave elastography (SWE) and hepatic inflammation/fibrosis stage, functional hepatic reserve, complications associated with chronic liver disease. **a** Correlation of hepatic Vs values measured by SWE in 71 patients (varices; *n* = 45) with liver disease with age, gender, BMI, serum liver function and fibrosis marker values, histological inflammation, and fibrosis stage, functional liver reserve, and portal hypertension-related complications. Data on gender, biopsy (A; grading, F; staging), Child–Pugh grade, ascites, and varices were assessed using the Mann–Whitney U test and represent median and interquartile range. Age, BMI, Plt, INR, T-bil, AST, ALT, γGTP, ALP, Alb, hyaluronic acid, type IV collagen 7S, M2BPGi, FIB-4 index, and splenic volume; correlations were evaluated using Spearman's rank correlation coefficient. The correlation coefficients obtained are listed respectively. **b**, **c** *p* values (-log2 ratio) in statistical analysis of Vs value (**b**) and histological fibrosis stage assessed in liver biopsy specimens (**c**) with each factor. *p* = 0.05 is indicated by a dotted line. Each data was divided between the two groups at the stated cutoff values and evaluated with the Mann–Whitney U test

Vs value was measured with pSWE, and liver biopsy specimens were collected from 71 patients with liver disease and investigated for correlation with function and complications associated with CLD (Fig. 1a and Table 1). The Vs value was highly correlated with fibrosis-related markers such as platelet count, serum hyaluronic acid, type IV collagen 7S, M2BPGi, FIB-4 index, and histological fibrosis stage and inflammation-related factors; AST and histological inflammation grade (Fig. 1b). Varices, a complication related to portal hypertension, and splenic volume also showed high association with Vs values. In addition, the stage of histological fibrosis in liver biopsies was assessed to significantly correlate with the fibrosis markers shown in Fig. 1c. Compared to the Vs values measured by pSWE, the histological fibrosis stage in liver biopsies showed no correlation with serum hyaluronic acid, FIB-4 index, Child–Pugh grade, and splenic volume. These results showed that the Vs values measured with pSWE were highly correlated with the clinical fibrosis-related markers in patients with chronic liver disease.

### Correlation between Vs value and liver fibrosis

**Fig. 2:**
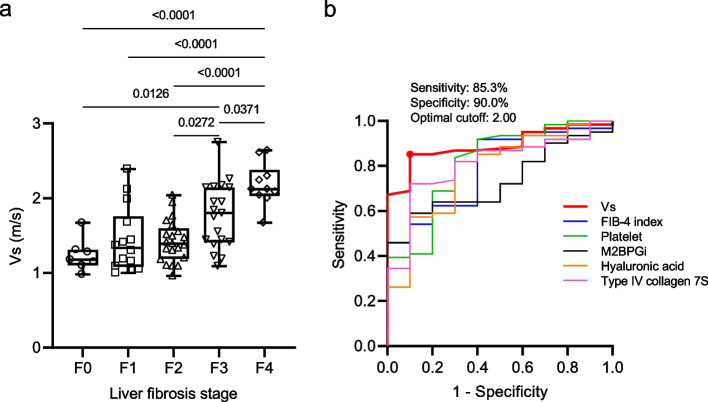
Predictive ability of shear wave elastography (SWE) for liver fibrosis compared with blood markers. **a** Box-and-whisker plots of shear wave velocity (Vs) values according to the degrees of liver fibrosis. The boxes denote the interquartile range (IQR) (i.e., 25th–75th percentiles) of shear wave measurement (SWM); the lines inside the boxes denote the medians (i.e., 50^th^ percentiles). Median (interquartile range) Vs values were measured in patients with fibrosis stage F0 (*n* = 7), F1 (*n* = 14), F2 (*n* = 21), F3 (*n* = 19), and F4 (*n* = 10). **b** Receiver-operating characteristic (ROC) curves for Vs values, FIB-4 index, platelet count, M2BPGi, hyaluronic acid, and type IV collagen 7S for the diagnosis of cirrhosis. The optimal cutoff value, sensitivity, and specificity when Vs values are used are described

We evaluated the relationship between the degree of histological fibrosis and Vs in 71 patients with liver disease. The median Vs was 1.18, 1.34, 1.39, 1.80, and 2.12 m/s in patients with F0, F1, F2, F3, and F4 stages of fibrosis, respectively (Fig. 2a). The Vs value increased with the progression of liver fibrosis. The predictive ability of platelet count, serum M2BPGi, hyaluronic acid, type IV collagen 7S, FIB-4 index, and Vs values for cirrhosis (F4) was compared using ROC analysis. The area under the receiver-operating characteristic curve (AUROC) was 0.902 (95%confidence interval [CI], 0.822–0.981) for the Vs value, 0.785 (95% CI, 0.641–0.929) for FIB-4 index, 0.812 (95% CI, 0.658–0.966) for platelet count, 0.730 (95% CI, 0.597–0.862) for M2BPGi, 0.784 (95% CI, 0.626–0.941) for hyaluronic acid, and 0.807 (95% CI, 0.675–0.94) for type IV collagen 7S (Fig. 2b). Comparison of ROC curves showed that AUROC for predicting cirrhosis of Vs value was not significantly different from AUROC for FIB-4 index, platelet count, hyaluronic acid and type IV collagen 7S, while it was significantly different from AUROC for M2BPGi (*P* < 0.01). The optimal cutoff value of Vs based on Youden index was 2.00 with a sensitivity of 85.3% and specificity of 90.0% (Fig. 2b). These results showed that Vs has a high correlation in predicting liver fibrosis.

### Correlation between Vs value and EVs

**Fig. 3:**
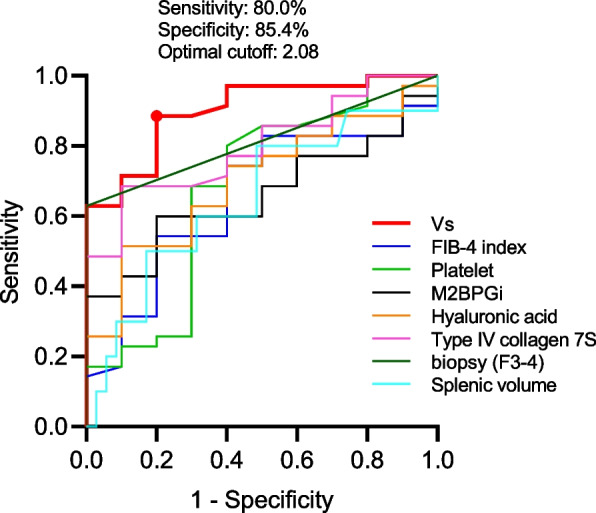
Predictive ability of shear wave elastography (SWE) for esophageal varices (EVs) compared with histological fibrosis stage, blood markers of liver fibrosis, and splenic volume. Receiver-operating characteristic (ROC) curves for Vs values, fibrosis stage 3–4 (liver biopsy), FIB-4 index, platelet count, serum levels of M2BPGi, hyaluronic acid, type IV collagen 7S, and splenic volume in predicting EVs. The optimal cutoff value, sensitivity, and specificity when Vs values are used are described

Of the 45 patients who underwent upper gastrointestinal endoscopy, EVs were identified in 10 patients (Table 1). The median Vs in the group without EVs was 1.44 m/s and that in the group with EVs was 2.21 m/s (*P* < 0.01) (Fig. 1a). The diagnostic value of Vs, histological fibrosis stage, blood markers, and splenic volume in predicting EVs was evaluated by ROC analysis (Fig. 3); the AUROC was 0.901 (95% CI, 0.805–1.000) for the Vs value, 0.814 (95% CI, 0.733–0.895) for fibrosis stage 3–4 (liver biopsy), 0.654 (95% CI, 0.466–0.843) for FIB-4 index, 0.680 (95% CI, 0.461–0.899) for platelet count, 0. 660 (95% CI, 0.496–0.824) for M2BPGi, 0.700 (95% CI, 0.529–0.871) for hyaluronic acid, 0.799 (95% CI, 0.662–0.935) for type IV collagen 7S, and 0.647 (95% CI, 0.431–0.863) for spleen volume. Comparison of ROC curves showed that AUROC for predicting EVs of Vs value was not significantly different from AUROC for fibrosis stage 3–4 (liver biopsy) and type IV collagen 7S, while it was significantly different from AUROC for FIB-4 index (*P* < 0.05), platelet count (*P* < 0.05), M2BPGi (*P* < 0.01), hyaluronic acid (*P* < 0.05) and splenic volume (*P* < 0.05). The optimal cutoff value of Vs based on Youden index was 2.08 with a sensitivity of 80.0% and specificity of 85.4%. These results showed that Vs is highly useful for predicting EVs.

### Vs values highly correlated with EVs in patients with advanced liver fibrosis

**Fig. 4:**
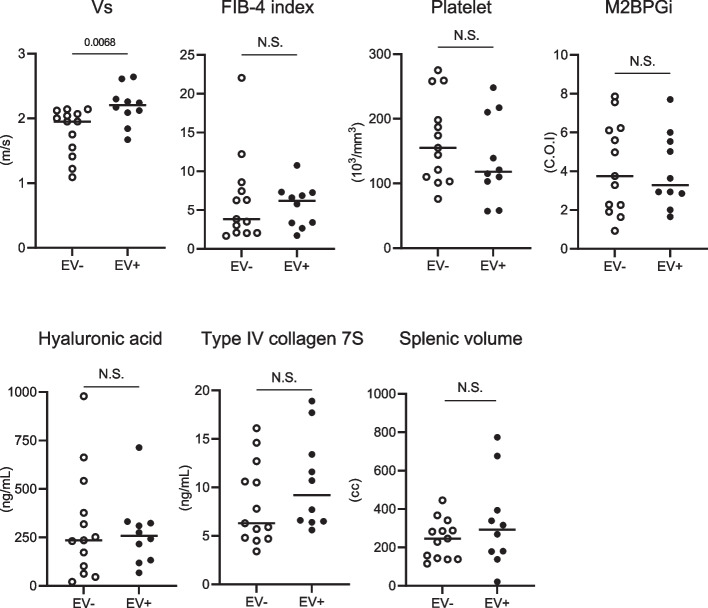
Vs values, blood markers, and splenic volume in the presence of esophageal varices (EVs) in patients with advanced liver fibrosis. Scatter plots of shear wave velocity (Vs) values, FIB-4 index, platelet count, M2BPGi, hyaluronic acid, type IV collagen 7S, and splenic volume in patients without (*n* = 13) and with (*n* = 10) esophageal varices (EVs) with advanced liver fibrosis. Bars represent medians for each group; N.S., not significantly different. Each plot shows Vs values, values of each blood marker of liver fibrosis, and spleen volume in individual patients. Each data set was assessed with the Mann–Whitney U test

We next evaluated factors reflecting the presence of EVs in patients with histologically advanced liver fibrosis (stages F3 and F4). The Vs values were significantly higher in the group with EVs than that in the group without EVs (Fig. 4). In contrast, the FIB-4 index, platelet count, serum levels of M2BPGi, hyaluronic acid, type IV collagen 7S, and splenic volume demonstrated no significant difference between the groups (Fig. 4). These results indicated that liver stiffness measurement by SWE may be more effective than blood markers and splenic volume for predicting the presence of EVs in patients with advanced liver fibrosis who are at high risk for EVs.

## Discussion

In the hepatic inflammation/fibrosis stage, functional hepatic reserve, and portal hypertension-related complications in patients with CLD, Vs values measured by pSWE were highly correlated with liver fibrosis and the complication rate of EVs. Vs values increased with the progression of liver fibrosis and tended to be a better predictor of cirrhosis (F4) than blood markers of liver fibrosis. Moreover, the diagnostic ability of Vs in predicting EVs was superior to blood markers and splenic volume. In advanced liver fibrosis patients, there was no difference in blood markers and splenic volume, while Vs value was high in patients with EVs.pSWE has been reported to be useful in diagnosing liver fibrosis caused by hepatitis B virus (HBV), hepatitis C virus (HCV), non-alcoholic steatohepatitis (NASH), alcoholic hepatitis, and autoimmune hepatitis (AIH) [18–22]. Numao et al*.* reported that two-dimensional (2D)-SWE is more useful in predicting liver fibrosis than FIB-4 index and M2BPGi [23]. Park et al*.* reported that pSWE is more useful in predicting liver fibrosis than FIB-4 index and aspartate aminotransferase to platelet ratio index (APRI) [21]. The reason for the superiority of SWE in its ability to diagnose liver fibrosis is that the amount of fibrous tissue is most accurately reflected by the hepatic elastic ratio determined by elastography [24]. These reports are consistent with our findings that SWE is superior to blood markers in diagnosing cirrhosis. While it should be noted that some patients have severe exacerbation with high ALT levels which are likely to affect the pSWE values, as suggested by the World Federation for Ultrasound in Medicine and Biology (WFUMB) and European Federation of Societies for Ultrasound in Medicine and Biology (EFSUMB) guidelines and recommendations [25–27].

In predicting the presence of EVs, correlation with blood tests, especially platelet counts and liver fibrosis markers, has been reported [28]. However, their AUROCs ranged from 0.57 to 0.77, which are not sufficiently accurate [28]. Vermehren et al*.* reported for the first time that liver stiffness measured by SWE is useful in predicting EVs using the Acuson S2000 (Siemens Healthcare, Erlangen, Germany) [29]. Since then, it has been reported that pSWE is useful in various liver diseases [30–32]. Portal hypertension, the underlying pathology of EVs, is defined as an increase in hepatic venous pressure gradient (HVPG) [33], Carrión et al*.* reported that liver stiffness is closely and directly correlated with HVPG [34]. In patients with advanced liver fibrosis diagnosed using liver biopsy, only SWE was useful in predicting EVs [33], which suggests that SWE better reflects the changes in HVPG.

In this study, liver stiffness correlated with histological liver fibrosis and esophageal varices compared to splenic volume. Many studies suggested that the diagnostic value of spleen elasticity for high-risk varices (HRV)/ clinically significant portal hypertension (CSPH) is superior to liver elasticity [35, 36]. Meanwhile, it has also been reported that spleen elasticity does not correlate with splenic volume [37]. Splenomegaly is a common finding in the natural history of patients with cirrhosis and portal hypertension [35]. Berzigotti et al*.* reported that the spleen size measured by ultrasound examinations was not superior to liver stiffness in the diagnosis of CSPH and varices [38]. In patients with cirrhosis, the splenic volume varies depending on the etiology [39] and has been influenced by portosystemic collaterals in the cases of advanced liver diseases [40, 41]. These findings support the results of the current study that Vs value is superior to splenic volume for diagnosing varices in cases with advanced liver fibrosis.

There are several limitations to this study. First, the sample size was small. One of the aims of this study was to assess the significance and correlation of pSWE in patients with the chronic liver disease based on a histological assessment of the degree of liver fibrosis and inflammation. The usefulness of pSWE has been assessed in cases in which a liver biopsy, the gold standard to assess the nature and severity of liver disease, was performed, but the number of cases was therefore limited. Second, this study was conducted at a single institution. Third, the pathogenesis of CLD is heterogeneous, which is likely to affect the relationship of pSWE values to fibrosis or EVs. Herrmann et al*.* also reported that the diagnostic performance of SWE for liver fibrosis differs for each etiology of liver disease [42], and the predictive performance of EVs is also expected to vary depending on the cause of liver disease. In the future, it may be important to increase the number of samples and perform analyses for each etiology of liver disease.

## Conclusions

The hepatic shear wave velocity of pSWE is highly correlated with liver fibrosis and the complication rate of EVs in patients with CLD. Furthermore, in patients with advanced liver fibrosis, the Vs value was more accurate than blood markers and splenic volume in predicting EVs. In patients with CLD, a non-invasive test using Vs values of pSWE was suggested to be useful as a screening test for predicting cirrhosis and EVs.

## Data Availability

The datasets used and/or analysed during the current study are available from the corresponding author on reasonable request.

## Abbreviations

2D: Two-dimensional
AIH: Autoimmune hepatitis
ALP: Alkaline phosphatase
ALT: Alanine aminotransferase
APRI: Aspartate aminotransferase to platelet ratio index
AST: Aspartate aminotransferase
AUC: Area under the curve
AUROC: Area under the receiver-operating characteristic curve
CLD: Chronic liver disease
CSPH: Clinically significant portal hypertension
CT: Computed tomography
EFSUMB: European Federation of Societies for Ultrasound in Medicine and Biology
EVs: Esophageal varices
HBV: Hepatitis B virus
HCV: Hepatitis C virus
HRV: High-risk varices
HVPG: Hepatic venous pressure gradient
INR: International normalized ratio
IQR: Interquartile range
M2BPGi: Mac-2 binding protein glycosylation isomer
NASH: Non-alcoholic steatohepatitis
pSWE: Point shear wave elastography
ROC: Receiver-operating characteristic
ROI: Region of interest
SWE: Shear wave elastography
SWM: Shear wave measurement
Vs: Shear wave velocity
WFUMB: World Federation for Ultrasound in Medicine and Biology
